# Cantharidal Strangury

**Published:** 1875-06

**Authors:** L. E. Starr

**Affiliations:** Bibb County, Ala.


### CANTHARIDAL STRANGURY.

By L. E. STARR, M.D., of Bibb County, Ala.

It is a well-known fact that strangury often results from the absorption of the active principles of the fly in the use of this cerate as a blistering agent; and to avoid which would disarm this great therapeutic agent of its only power to do mischief when properly used.

The object of this short article is to report the result of my experience and observation for the past twelve years on this subject. I, as well as many others, being hard pressed for good and fresh drugs during the late war, was forced to use old and inferior articles, and in using some old cerate I was often disappointed because I did not get a good blister, and in order to get it to act well I used various substances to assist it, such as oil of turpentine, tincture of capsicum, alcohol, etc. With the plaster moistened with any of these I could get a good blister, alcohol being best. For a time I supposed this to be all the advantage from its use, but after a time my attention was called to the fact that I never had strangury while using it, and since then I have continued to moisten my plasters, just before applying, with alcohol, no matter how fresh was my cerate, and I have never had a single case of strangury.

Any attempt at an explanation of the *modus operandi* of the action of alcohol in the prevention of strangury would be only speculation, as its virtues have not been sufficiently tested. The experience of others may prove that it is worthless; yet it has seemed to me to prevent the occurrence of strangury with great uniformity.

